# Supplementary material for: In vitro evidence to support amphotericin B and flucytosine combination therapy for talaromycosis
Source: PLoS Negl Trop Dis. 2025 Dec 31;19(12):e0013884. doi: 10.1371/journal.pntd.0013884 (PMC12782420; doi:10.1371/journal.pntd.0013884)
Supplement: S1 Appendix — (DOCX) [file pntd.0013884.s001.docx]

**S1 Appendix: Additional methods information**

***Talaromyces marneffei* isolates**

The clinical isolates were revived on yeast peptone dextrose (YPD) agar plates and grown in the yeast form at 37°C. After 3 to 6 days, a single colony was sub-cultured on a new YPD plate to achieve purity. Pure yeast cultures were maintained at 4°C for up to 2 weeks and sub-cultured at 37°C for 3 days for antifungal susceptibility experiments.

**Antifungal drug preparation**

Drug stocks, drug dilutions, and drug plates were prepared according to the Clinical and Laboratory Standards Institute (CLSI) guidelines [1]. AmB and 5FC (Sigma-Aldrich, St. Louis, MO, United States) were obtained as pure powders. In brief, AmB was dissolved in dimethyl sulfoxide (DMSO), and 5FC was dissolved in Roswell Park Memorial Institute (RPMI) medium (Sigma-Aldrich, United States) buffered to pH 7 with 3-(N-morpholino) propane sulfonic acid (MOPS, Sigma-Aldrich, United States). Serial dilutions of AmB and 5FC were prepared in RPMI-MOPS, and aliquots were stored at -20°C and thawed to room temperature before use. Our previous study reported MIC ranges of 0.5 – 1 µg/mL for AmB (95% inhibition) and 0.06 – 0.5 µg/mL for 5FC (50% inhibition) [2]. Based on these findings, the final drug concentration ranges tested were 0.03 – 2 μg/mL for AmB and 0.004 – 2 µg/mL for 5FC.

**Antifungal susceptibility testing by our validated CLSI-based colorimetric assay**

In brief, on the day of inoculation, *T. marneffei* yeast cells were harvested from the YPD plates and suspended in phosphate-buffered saline (PBS). Using an Ultrospec^®^ cell density meter (Biochrom, Holliston, MA, United States), the inoculum was standardized to an optical density at 600 nm (OD_600nm_) range of 0.52 – 0.58, which is equivalent to 1 – 5 x 10^6^ CFU/mL. The inoculum was further diluted in RPMI-MOPS, which resulted in a working inoculum of 1 – 5 x 10^3^ CFU/mL. A total of 100µL of inoculum was added to each well of the prepared drug plate, excluding the negative control well. The plates were sealed and incubated at 37°C. After 24 hours of incubation, alamarBlue was added to all wells, and the plates were incubated at 37°C for an additional 48 hours. Following incubation, FI was measured at an excitation of 570 nm and emission of 590 nm using a fluorescence spectrophotometer (BMG LABTECH, Cary, NC, United States). The percentage reduction of fungal growth was calculated for each well in reference to the positive and negative control well.

**Equation A for calculating the fractional inhibitory concentration index (FICI) by checkerboard assay**

$$\left( \mathbf{A} \right) FICI= \frac{{MIC}_{amphotericin B in combination}}{{MIC}_{amphotericin B alone}}+ \frac{{MIC}_{flucytosine in combination}}{{MIC}_{flucytosine alone}}$$

**Time-kill assay**

In brief, a standardized *T. marneffei* yeast inoculum of 10^5^ cells/mL was prepared with PBS. The MIC ranges of AmB and 5FC were determined using our CLSI-based colorimetric broth microdilution method [2]. Dose-response curves were generated using AmB at 0.25, 0.5, 1, and 2 times the MIC, and for 5FC at 0.25, 0.5, 1, 5, 10, and 20 times the MIC, reflecting clinically achievable drug levels in plasma [3,4]. For AmB and 5FC combination testing, sub-MIC concentrations of AmB (0.25 and 0.5 times the MIC) were used, as these were concentrations that were not rapidly fungicidal (as determined by the time-kill experiment for AmB alone) and thus permitted accurate assessment of the added effect of 5FC, which was tested at concentrations 1, 5, 10, and 20 times the MIC. Ten-milliliter tubes were prepared with the fungal suspension, antifungal drugs (alone or in combination), and RPMI-MOPS. A control tube containing no drug was included in all experiments. The tubes were incubated at 37°C at 250 rpm. At each pre-determined timepoint (24, 48, 72, 96, and 120 hours), 0.1 – 0.3 mL was aliquoted from each testing condition, diluted appropriately, and plated on YPD in duplicate. Fungal growth was measured by the colony forming units (CFUs/mL).

**Quality control**

Drug concentrations were standardized based on the expected MIC of the *Candida krusei* ATCC 6258 reference strain, according to CLSI methods. *C. krusei* ATCC 6258 was included as a quality control in all susceptibility experiments, alongside *T. marneffei* strain 11CN-20-091 as an internal control. On each day of inoculation, the final *T. marneffei* inoculum was grown on YPD plates at 37°C for 5 days to check the viability of the inoculum used. A CFU count within the range of 5 – 30 was considered optimal.

**References**

1. M27-A3: Reference Method for Broth Dilution Antifungal Susceptibility Testing of Yeasts; Approved Standard—Third Edition.

2. Thu NTM, Borda H, Vitsupakorn S, et al. Development and validation of a colorimetric antifungal susceptibility testing method for the dimorphic fungus Talaromyces marneffei. Med Mycol **2023**; 61:myad111.

3. Sigera LSM, Denning DW. Flucytosine and its clinical usage. Therapeutic Advances in Infection **2023**; 10:20499361231161387.

4. Stott KE, Beardsley J, Whalley S, et al. Population Pharmacokinetic Model and Meta-analysis of Outcomes of Amphotericin B Deoxycholate Use in Adults with Cryptococcal Meningitis. Antimicrob Agents Chemother **2018**; 62:e02526-17.
