# Supplementary material for: In vitro evidence to support amphotericin B and flucytosine combination therapy for talaromycosis
Source: PLoS Negl Trop Dis. 2025 Dec 31;19(12):e0013884. doi: 10.1371/journal.pntd.0013884 (PMC12782420; doi:10.1371/journal.pntd.0013884)
Supplement: S1 Table — (DOCX) [file pntd.0013884.s002.docx]

**S1 Table**

**Distribution of the minimum inhibitory concentrations and combination effect between amphotericin B and flucytosine for 60 *Talaromyces marneffei* clinical isolates.**

| **Interaction Type** | **MIC_95_ (µg/mL)** | | **FIC _AmB_** | **MIC_95_ (µg/mL)** | | | **FIC _5FC_** | **FICI**  FIC_AmB_+FIC_5FC_ |
| --- | --- | --- | --- | --- | --- | --- | --- | --- |
|  | **AmB** | **AmB+5FC** |  | **5FC** | **5FC+AmB** | |  |  |
| ***Synergy*** *(FICI ≤ 0.5), n = 4* | | | | | | | | |
| 11CN-21-012 | 2.00 | 0.50 | 0.25 | 1.00 | | 0.06 | 0.06 | 0.31 |
| 11CN-21-040 | 1.00 | 0.25 | 0.25 | 0.13 | | 0.02 | 0.13 | 0.38 |
| 11CN-27-012 | 2.00 | 0.50 | 0.25 | 0.50 | | 0.06 | 0.13 | 0.38 |
| 11CN-27-017 | 2.00 | 0.50 | 0.25 | 0.25 | | 0.06 | 0.25 | 0.50 |
| ***Indifference*** *(0.5 < FICI ≤ 4), n = 56* | | | | | | | | |
| 11CN-03-002 | 0.50 | 0.25 | 0.50 | 0.50 | | 0.13 | 0.25 | 0.75 |
| 11CN-03-006 | 1.00 | 0.50 | 0.50 | 0.25 | | 0.00 | 0.02 | 0.52 |
| 11CN-03-007 | 0.25 | 0.02 | 0.06 | 0.25 | | 0.25 | 1.00 | 1.06 |
| 11CN-03-008 | 0.25 | 0.03 | 0.13 | 0.13 | | 0.13 | 1.00 | 1.13 |
| 11CN-03-009 | 0.50 | 0.50 | 1.00 | 0.25 | | 0.02 | 0.06 | 1.06 |
| 11CN-03-014 | 0.25 | 0.13 | 0.50 | 0.50 | | 0.13 | 0.25 | 0.75 |
| 11CN-03-015 | 0.50 | 0.13 | 0.25 | 1.00 | | 0.50 | 0.50 | 0.75 |
| 11CN-03-021 | 0.50 | 0.02 | 0.03 | 0.50 | | 0.25 | 0.50 | 0.53 |
| 11CN-03-029 | 1.00 | 0.50 | 0.50 | 0.50 | | 0.06 | 0.13 | 0.63 |
| 11CN-03-037 | 0.25 | 0.13 | 0.50 | 0.25 | | 0.13 | 0.50 | 1.00 |
| 11CN-03-039 | 0.25 | 0.13 | 0.50 | 1.00 | | 0.50 | 0.50 | 1.00 |
| 11CN-03-040 | 0.25 | 0.06 | 0.25 | 1.00 | | 0.50 | 0.50 | 0.75 |
| 11CN-03-048 | 0.25 | 0.13 | 0.50 | 1.00 | | 0.50 | 0.50 | 1.00 |
| 11CN-03-051 | 0.50 | 0.25 | 0.50 | 0.25 | | 0.13 | 0.50 | 1.00 |
| 11CN-03-068 | 1.00 | 1.00 | 1.00 | 0.25 | | 0.00 | 0.02 | 1.02 |
| 11CN-03-070 | 1.00 | 0.50 | 0.50 | 0.25 | | 0.06 | 0.25 | 0.75 |
| 11CN-03-078 | 0.25 | 0.25 | 1.00 | 0.50 | | 0.02 | 0.03 | 1.03 |
| 11CN-03-083 | 0.50 | 0.25 | 0.50 | 0.25 | | 0.06 | 0.25 | 0.75 |
| 11CN-03-086 | 1.00 | 0.50 | 0.50 | 0.25 | | 0.03 | 0.13 | 0.63 |
| 11CN-03-098 | 2.00 | 1.00 | 0.50 | 0.50 | | 0.01 | 0.02 | 0.52 |
| 11CN-03-104 | 0.50 | 0.25 | 0.50 | 0.50 | | 0.25 | 0.50 | 1.00 |
| 11CN-03-108 | 1.00 | 1.00 | 1.00 | 0.13 | | 0.00 | 0.03 | 1.03 |
| 11CN-03-116 | 1.00 | 0.50 | 0.50 | 0.25 | | 0.13 | 0.50 | 1.00 |
| 11CN-03-120 | 1.00 | 0.50 | 0.50 | 0.25 | | 0.06 | 0.25 | 0.75 |
| 11CN-03-121 | 1.00 | 0.06 | 0.06 | 0.25 | | 0.13 | 0.50 | 0.56 |
| 11CN-03-129 | 1.00 | 0.50 | 0.50 | 0.25 | | 0.06 | 0.25 | 0.75 |
| 11CN-03-130 | 0.50 | 0.25 | 0.50 | 0.50 | | 0.25 | 0.50 | 1.00 |
| 11CN-03-140 | 1.00 | 0.50 | 0.50 | 0.13 | | 0.06 | 0.50 | 1.00 |
| 11CN-03-147 | 1.00 | 0.06 | 0.06 | 0.50 | | 0.25 | 0.50 | 0.56 |
| 11CN-03-148 | 1.00 | 0.25 | 0.25 | 0.06 | | 0.03 | 0.50 | 0.75 |
| 11CN-03-153 | 1.00 | 0.50 | 0.50 | 0.25 | | 0.00 | 0.02 | 0.52 |
| 11CN-03-154 | 0.50 | 0.25 | 0.50 | 0.06 | | 0.03 | 0.50 | 1.00 |
| 11CN-03-158 | 1.00 | 0.50 | 0.50 | 0.50 | | 0.03 | 0.06 | 0.56 |
| 11CN-20-002 | 1.00 | 0.50 | 0.50 | 0.25 | | 0.06 | 0.25 | 0.75 |
| 11CN-20-005 | 0.50 | 0.25 | 0.50 | 0.50 | | 0.25 | 0.50 | 1.00 |
| 11CN-20-008 | 1.00 | 0.50 | 0.50 | 0.50 | | 0.25 | 0.50 | 1.00 |
| 11CN-20-016 | 1.00 | 0.03 | 0.03 | 0.25 | | 0.13 | 0.50 | 0.53 |
| 11CN-20-023 | 0.50 | 0.25 | 0.50 | 0.13 | | 0.06 | 0.50 | 1.00 |
| 11CN-20-029 | 0.50 | 0.02 | 0.03 | 0.13 | | 0.13 | 1.00 | 1.03 |
| 11CN-20-044 | 0.50 | 0.25 | 0.50 | 0.13 | | 0.03 | 0.25 | 0.75 |
| 11CN-20-052 | 1.00 | 0.50 | 0.50 | 2.00 | | 0.06 | 0.03 | 0.53 |
| 11CN-20-065 | 0.50 | 0.50 | 1.00 | 0.25 | | 0.02 | 0.06 | 1.06 |
| 11CN-20-068 | 1.00 | 0.50 | 0.50 | 0.25 | | 0.06 | 0.25 | 0.75 |
| 11CN-20-070 | 1.00 | 0.50 | 0.50 | 1.00 | | 0.13 | 0.13 | 0.63 |
| 11CN-20-078 | 1.00 | 0.03 | 0.03 | 0.50 | | 0.25 | 0.50 | 0.53 |
| 11CN-20-079 | 0.50 | 0.25 | 0.50 | 0.06 | | 0.03 | 0.50 | 1.00 |
| 11CN-20-091 | 0.25 | 0.13 | 0.50 | 0.25 | | 0.03 | 0.13 | 0.63 |
| 11CN-20-102 | 1.00 | 0.50 | 0.50 | 0.25 | | 0.13 | 0.50 | 1.00 |
| 11CN-20-120 | 1.00 | 0.50 | 0.50 | 0.25 | | 0.03 | 0.13 | 0.63 |
| 11CN-26-003 | 0.50 | 0.13 | 0.25 | 0.06 | | 0.03 | 0.50 | 0.75 |
| 11CN-21-013 | 2.00 | 1.00 | 0.50 | 0.13 | | 0.00 | 0.03 | 0.53 |
| 11CN-21-022 | 1.00 | 0.50 | 0.50 | 0.25 | | 0.03 | 0.13 | 0.63 |
| 11CN-21-028 | 2.00 | 1.00 | 0.50 | 1.00 | | 0.02 | 0.02 | 0.52 |
| 11CN-21-041 | 0.25 | 0.13 | 0.50 | 0.06 | | 0.02 | 0.25 | 0.75 |
| 11CN-26-024 | 0.50 | 0.25 | 0.50 | 0.06 | | 0.02 | 0.25 | 0.75 |
| 11CN-27-009 | 0.25 | 0.13 | 0.50 | 0.06 | | 0.03 | 0.50 | 1.00 |
| Mean | 0.68 | 0.24 | 0.45 | 0.28 | | 0.06 | 0.33 | 0.77 |
|  | (95% CI: 0.58-0.80) | (95% CI: 0.19-0.32) | (0.23) | (95% CI: 0.22-0.34) | | (95% CI: 0.04-0.08) | (0.25) | (0.22) |
| Mode | 1 | 0.5 | 0.5 | 0.25 | | 0.06 | 0.5 | 0.75 |

The MICs of AmB and 5FC were defined as the lowest drug concentration that resulted in at least 95% inhibition of fungal growth. The geometric means with the 95% confidence intervals are reported for the MICs of AmB and 5FC, alone, and in combination. The arithmetic means with the standard deviations are reported for FICs of AmB and 5FC, and the FICI for 60 isolates.

Abbreviations: 5FC, flucytosine; 95% CI, 95% confidence interval; AmB, amphotericin B; FIC, fractional inhibitory concentration; FICI, fractional inhibitory concentration index; MIC, minimum inhibitory concentration.
