# Supplementary material for: In vitro evidence to support amphotericin B and flucytosine combination therapy for talaromycosis
Source: PLoS Negl Trop Dis. 2025 Dec 31;19(12):e0013884. doi: 10.1371/journal.pntd.0013884 (PMC12782420; doi:10.1371/journal.pntd.0013884)
Supplement: Attachment — Submitted filename: Response to Reviewers_AmB-5FC_talaromycosis.docx [file pntd.0013884.s005.docx]

**Rebuttal Letter**

**Manuscript ID: PNTD-D-25-01840**

**Manuscript title: *In Vitro* Evidence to Support Amphotericin B and Flucytosine Combination Therapy for Talaromycosis**

**To: Editorial Office,** PLOS Neglected Tropical Diseases

**Re:** Response to Reviewer

December 5, 2025

Dear Editors and Reviewers,

We appreciate your comprehensive review and valuable feedback of our manuscript. We have considered the comments and revised the manuscript accordingly. Please find below our point-by-point responses to your comments.

Sincerely,

Heera Natesan Sambath, MBBS

Thuy Le, MD, DPhil

**Reviewer #1**:

1. The text states: "Conversely, AmB is a large molecule that has variable penetration in the lungs, liver, kidneys, and a poor penetration in the brain and CSF (30,31). The disruption of pyrimidine biosynthesis by 5FC leads to structural defects in the fungal cell wall, facilitating membrane and tissue penetration of AmB and potentiating the fungicidal effect of AmB (29)."(Line 323-327). The current phrasing, particularly the use of "Conversely... facilitating... tissue penetration of AmB", could be misinterpreted. The reason is: The "tissue penetration of AmB" (e.g., in lungs, brain) is a pharmacokinetic concept, describing the drug's distribution in human organs and across barriers like the blood-brain barrier. The “disruption of the fungal cell wall by 5FC" is a pharmacodynamic concept, describing the drug's effect on the pathogen's microstructure. Linking them in this way might incorrectly suggest that the damage to the fungal cell wall by 5FC enhances the penetration of AmB through human tissues.

**Response:** We thank the reviewer for this valid point. We agree that our previous text linking pharmacodynamic effect of combination therapy on the pathogen to the improvement of the pharmacokinetics of the drug in the host is confusing. We have revised the paragraph in lines 339 to 347 to address this point, as follows:

*“Conversely, 5FC disrupts of the pyrimidine biosynthesis, leading to structural defects in the fungal cell wall, thereby potentiating the fungicidal effect of AmB (29). A major pharmacokinetic limitation of AmB is its large molecular size with variable penetration in the lungs, liver, kidneys, and poor penetration in the brain and CSF (30,31). The synergistic effect of 5FC on AmB may compensate for the pharmacokinetic limitation of AmB, and improve the treatment of deep-seated and multi-organ dissemination nature of talaromycosis. Enhanced clearance of deep-seated infection may also reduce the incidence of disease relapse, and incidence of immune reconstitution disease as the immune system of the host is restored, such as during antiretroviral therapy in patients with HIV (18).”*

2. The paper notes that all four isolates showing full synergy were from northern Vietnam, raising the question of clade-specific treatment effects. Could the authors please discuss or speculate on the potential molecular mechanisms that might underlie this interesting observation? Furthermore, have similar geographically or phylogenetically clade-specific synergistic effects of antimicrobial combinations been reported in other important fungi, such as Candida or Cryptococcus species?

**Response:** We thank you for another excellent point. In response, we have added a new paragraph in the discussion entitled “*Clade-specific difference in antifungal susceptibility*” where we went deeper in the clade-specific antifungal effects in *T. marneffei* and other fungi on lines 366 to 401, as follows.

*“Clade-specific difference in antifungal susceptibility*

*Interestingly, all four isolates showing full synergy were from northern Vietnam, raising the question of whether antifungal effect is clade-specific (3). We have previously shown, in a genome wide association study of the 336 human T. marneffei isolates collected from the IVAP trial patients, that isolates from northern and southern Vietnam form two distinct geographical and phylogenetic clades (3). Although we observed no significant differences in the MICs of AmB or itraconazole between the two clades nor clade-specific treatment responses, there were significant associations between the southern clade and biomarkers of disease severity, i.e., higher median baseline fungal load (2.5 vs 2.1 log_10_ CFU/mL, P = 0.024, by Wilcoxon rank sum test) and absence of fever (34% vs 10%, P = 0.0002, by Fisher’s exact probability test) (3). Additionally, genomic analysis showed reduced diversity in the southern clade, suggesting that it represents a more recently evolved lineage (3). Clade-specific differences in azole resistance levels have been documented for other WHO fungal priority pathogens: Candidozyma (formerly Candida) auris and Cryptococcus neoformans (38–41), suggesting that variation in antifungal combination activity is plausible. However, to our knowledge, no studies have systematically compared antifungal combination activity between genetic clades of yeasts, molds, or dimorphic fungi. Previous studies of antifungal combinations in these species either do not stratify outcomes by clades or lack direct comparisons between clades (39,42,43), and warrant further investigations.”*

**New references that were added from this new paragraph are:**

38.	Lockhart SR, Etienne KA, Vallabhaneni S, Farooqi J, Chowdhary A, Govender NP, et al. Simultaneous Emergence of Multidrug-Resistant Candida auris on 3 Continents Confirmed by Whole-Genome Sequencing and Epidemiological Analyses. Clin Infect Dis. 2017 Jan 15;64(2):134–40.

39.	John LLH, Thomson DD, Bicanic T, Hoenigl M, Brown AJP, Harrison TS, et al. Heightened Efficacy of Anidulafungin When Used in Combination with Manogepix or 5-Flucytosine against Candida auris In Vitro. Antimicrob Agents Chemother. 2023 May 10;67(6):e01645-22.

40.	Chong HS, Dagg R, Malik R, Chen S, Carter D. In Vitro Susceptibility of the Yeast Pathogen Cryptococcus to Fluconazole and Other Azoles Varies with Molecular Genotype. J Clin Microbiol. 2010 Nov;48(11):4115–20.

41.	Tomazin R, Matos T, Meis JF, Hagen F. Molecular Characterization and Antifungal Susceptibility Testing of Sequentially Obtained Clinical Cryptococcus deneoformans and Cryptococcus neoformans Isolates from Ljubljana, Slovenia. Mycopathologia. 2018 Apr 1;183(2):371–80.

42.	Nagy F, Tóth Z, Nyikos F, Forgács L, Jakab Á, Borman AM, et al. In vitro and in vivo interaction of caspofungin with isavuconazole against Candida auris planktonic cells and biofilms. Med Mycol. 2021 Oct 1;59(10):1015–23.

43.	Pfaller MA, Messer SA, Deshpande LM, Rhomberg PR, Utt EA, Castanheira M. Evaluation of Synergistic Activity of Isavuconazole or Voriconazole plus Anidulafungin and the Occurrence and Genetic Characterization of Candida auris Detected in a Surveillance Program. Antimicrob Agents Chemother. 2021 Mar 18;65(4):10.1128/aac.02031-20.
